# Olmesartan‐induced gastritis with no lower gastrointestinal symptoms: A case report

**DOI:** 10.1002/deo2.70124

**Published:** 2025-04-29

**Authors:** Satoshi Kosaka, Miki Kamiyama, Masahiro Ochi

**Affiliations:** ^1^ Department of Gastroenterology Meijibashi Hospital Osaka Japan

**Keywords:** angiotensin II type 1 receptor blockers, antihypertensive agents, drug‐related side effects and adverse reactions, esophagogastroduodenoscopy, gastrointestinal hemorrhage

## Abstract

A 74‐year‐old man with decreased appetite, weight, and heartburn was referred to our hospital. His medications included olmesartan. Esophagogastroduodenoscopy (EGD) revealed antral‐dominant erosive gastritis and nodular mucosa. A gastric biopsy revealed inflammatory cell infiltration. The serum anti‐*Helicobacter pylori* immunoglobulin G antibody test result was negative. Famotidine was ineffective in relieving his symptoms, and esomeprazole failed to prevent overt gastric bleeding, which required endoscopic hemostasis. The working diagnosis was drug‐induced gastritis, particularly olmesartan‐induced gastritis. His appetite loss started to improve within a week of olmesartan withdrawal. The erosions healed on EGD 2 months later. Over the next 10 months, he remained in his usual state until olmesartan was inadvertently administered. Subsequent EGD revealed a mild gastritis relapse. We diagnosed olmesartan‐induced gastritis and discontinued olmesartan treatment. Mucosal healing was confirmed by EGD 1 year later. Olmesartan is known to cause angiotensin II receptor blocker‐induced enteropathy. Although angiotensin II receptor blocker‐induced enteropathy affects the stomach, angiotensin II receptor blocker‐induced gastritis without lower gastrointestinal symptoms is rare. The characteristic endoscopic appearance may provide a clue to the correct diagnosis.

## INTRODUCTION

Angiotensin II receptor blockers (ARBs), including olmesartan, are widely used as antihypertensive agents owing to their favorable effects on renal and cardiovascular protection. Olmesartan is also recognized as a cause of enteropathy; however, olmesartan‐induced gastritis without significant lower gastrointestinal symptoms has rarely been reported. Here, we report a case of olmesartan‐induced gastritis with a characteristic endoscopic appearance, in which re‐administration of olmesartan reproduced gastritis.

## CASE REPORT

**FIGURE 1:**
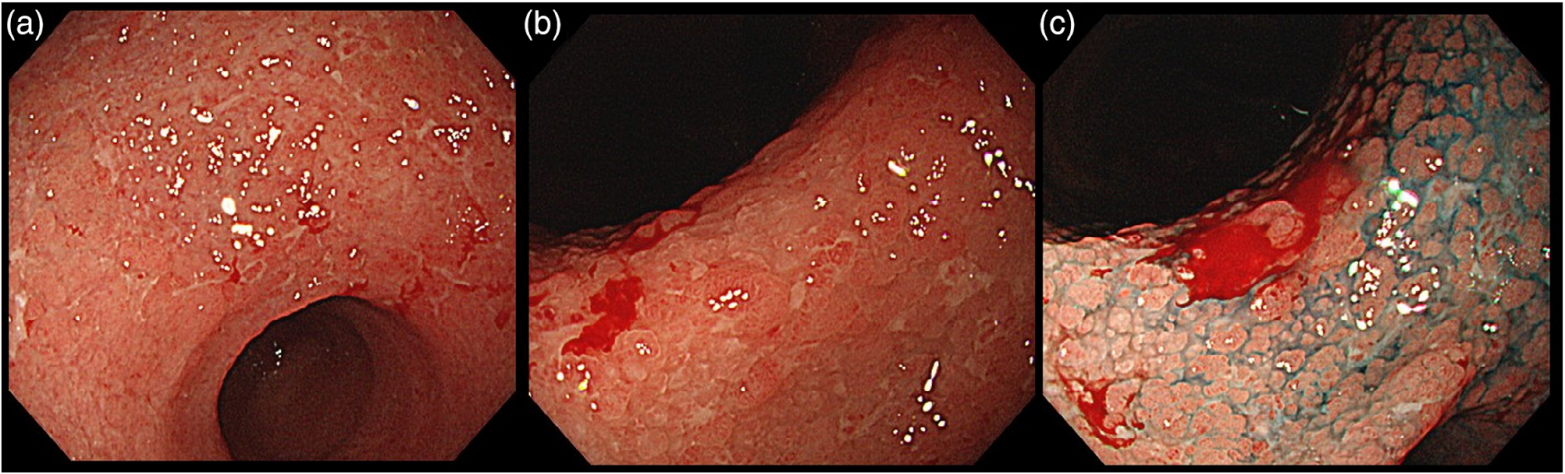
Erosive gastritis in the antrum. (a, b) Numerous small erosions clustered. Residual mucosa was erythematous and edematous. (c) Island of inflamed mucosa appeared cobblestone‐like, which was better observed with indigo carmine.

**FIGURE 2:**
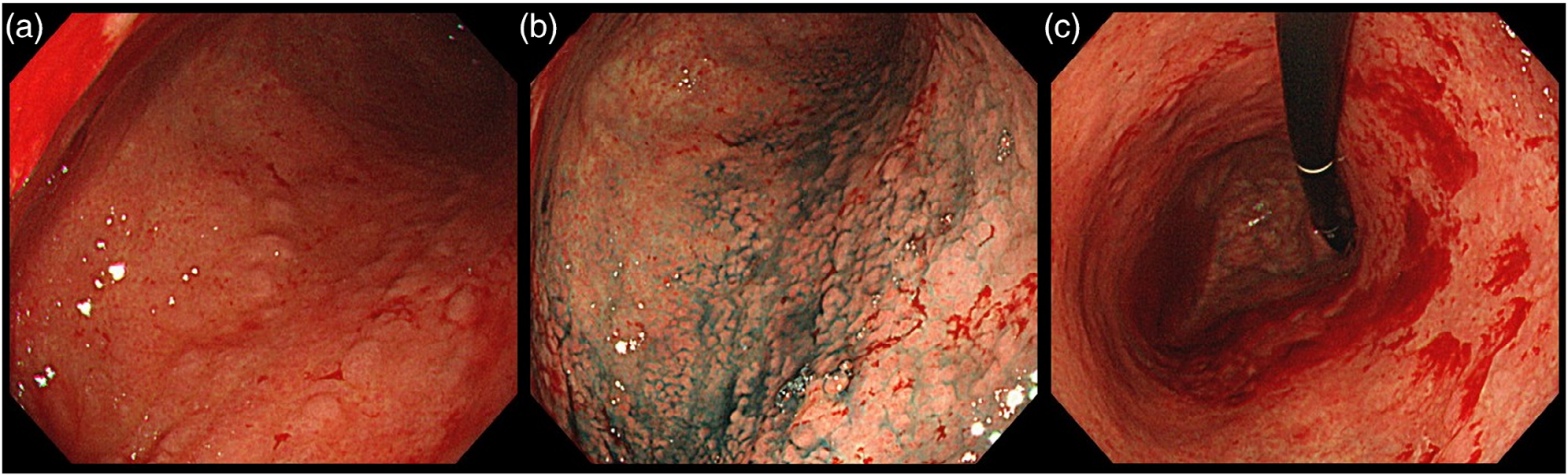
Nodular appearance in the corpus and friable mucosa. (a, b) Mucosa was mildly erythematous and edematous in the corpus. The nodular appearance was better observed with indigo carmine. (c) Mucosa was friable and bled with endoscopic contact.

**FIGURE 3:**
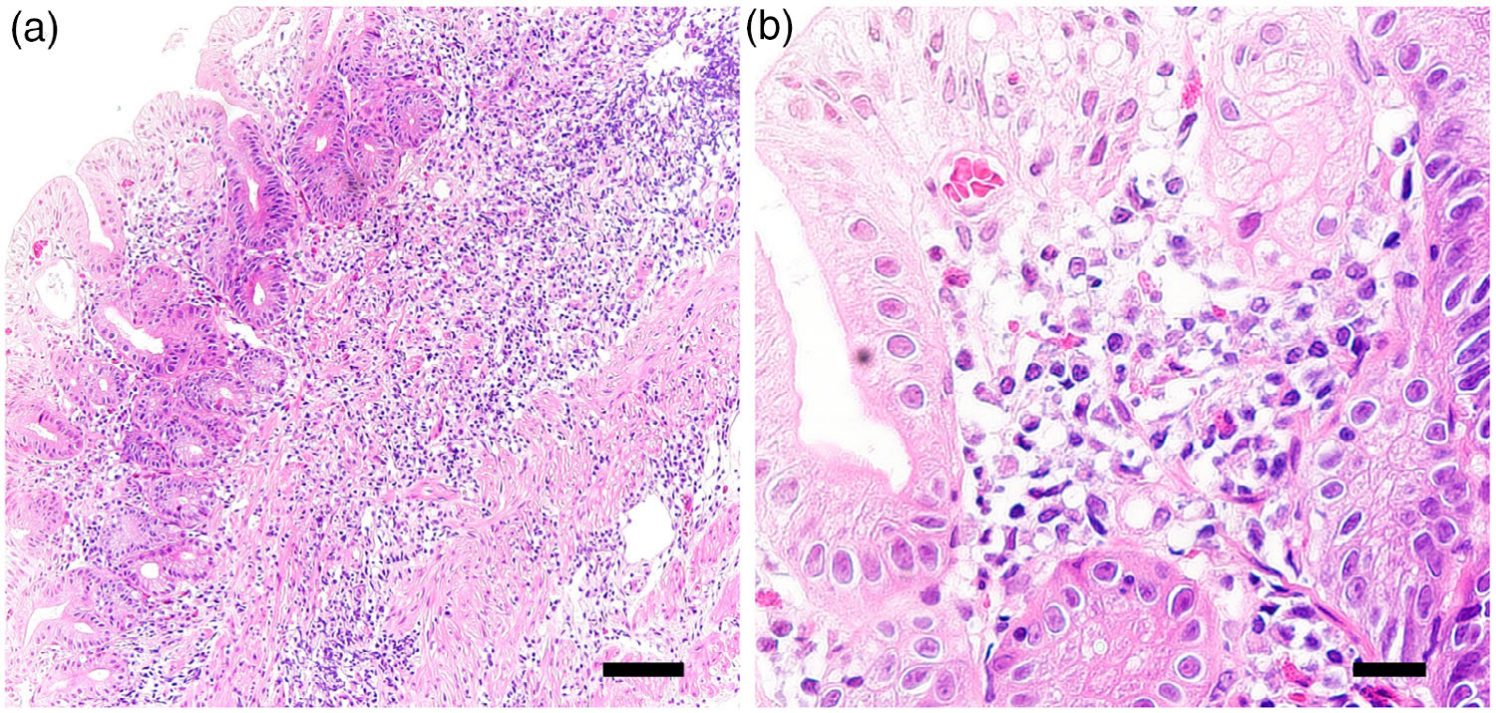
Inflammatory cell infiltration in the stomach. (a, b) Gastric biopsies revealed inflammatory cell infiltration with no atypical cells, subepithelial collagen deposition, or marked eosinophil infiltration (hematoxylin and eosin staining). The scale bars in (a) and (b) represent 100 and 20 µm, respectively.

**FIGURE 4:**
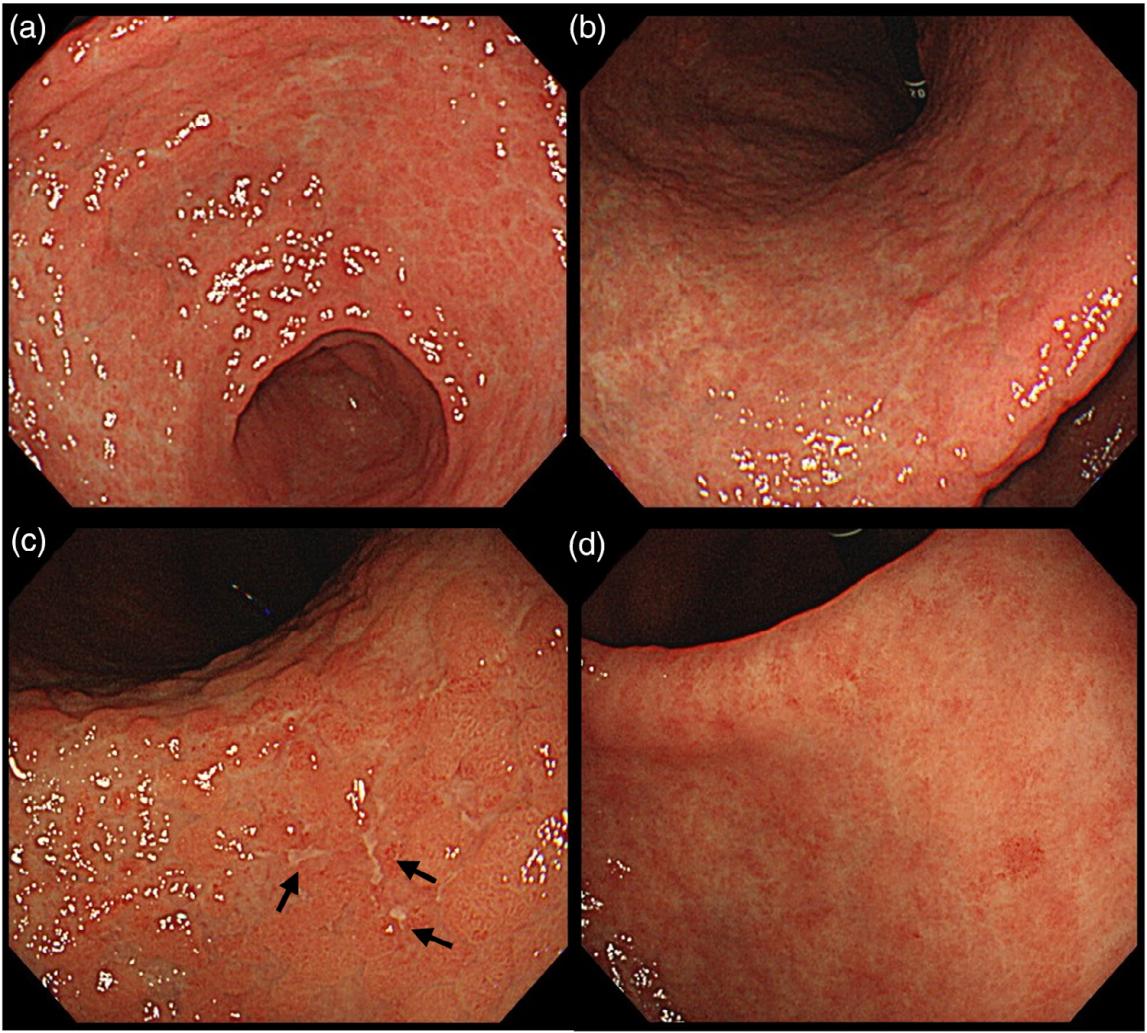
The relapse after re‐administration of olmesartan. (a, b) Erosion healed on esophagogastroduodenoscopy 2 months after olmesartan withdrawal. The mucosa was nodular. (c) Multiple short linear erosions developed after incidental re‐administration of olmesartan (arrows), but the lesions were minor compared to those observed in the initial esophagogastroduodenoscopy. Nodularity was more pronounced than that observed in (a). (d) Nodularity resolved when olmesartan was discontinued for 1 year.

A 74‐year‐old man with decreased appetite, weight, and heartburn was referred to our hospital. Six months prior to the current presentation, heartburn began, and 5 months later, he lost his appetite. His weight decreased by 7 kg from his usual weight of 64 kg, without fever or diarrhea. His medical history included well‐controlled diabetes mellitus, hypertension, constipation, pruritus, and gastric ulcer. His medications were olmesartan 20 mg daily for 13 years, miglitol 100 mg daily for 8 years, dapagliflozin 5 mg daily for 6 years, sennoside 24 mg daily for 2 months, and a combination of betamethasone 0.25 mg and d‐chlorpheniramine 0.4 mg daily for 3 months. Famotidine was administered to treat suspected acid‐related diseases. One week later, esophagogastroduodenoscopy (EGD) revealed numerous small erosions in the lesser curvature of the lower corpus and antrum (Figure 1a,b). The areas of inflamed mucosal islands interspersed by erosion had a cobblestone appearance (Figure 1b,c). The rest of the corpus lacked erosions but appeared nodular (Figure 2a,b). The gastric mucosa of the lesser curvature was friable and bled on contact with the endoscope (Figure 2c). The esophageal and duodenal mucosa were endoscopically normal, except for a scar in the duodenal bulb. These gastric mucosal changes were not observed in the EGD performed at a clinic a year prior to the current presentation (Figure S1). Five gastric biopsies were performed, revealing inflammatory cell infiltration without atypical cells (Figure 3a,b). The atrophy was moderate in the antrum and severe in the corpus. No subepithelial collagen deposition (Masson's trichrome stain), amyloid deposits (Congo red stain), or more than 20 eosinophils per high‐power field were identified. Laboratory studies were unrevealing, including a negative result for serum anti‐*Helicobacter pylori* immunoglobulin G antibody test (Table S1). Computed tomography and abdominal ultrasonography findings were unremarkable. As his symptoms persisted, famotidine was changed to esomeprazole and mosapride. Six days later, he was initially presented to the emergency department for hematemesis and melena but was subsequently transferred to the gastroenterology department for further evaluation and management. An EGD revealed a gastric Dieulafoy lesion with oozing bleeding in the lesser curvature of the middle corpus (Figure S2), which was not the site of the biopsies. Endoscopic hemostasis was successfully achieved, and he was admitted to our hospital. Intravenous lansoprazole and oral misoprostol were administered. As gastric bleeding was well‐controlled after endoscopic hemostasis, intravenous lansoprazole was switched to oral esomeprazole. The duodenal ulcer scar and his history of gastric ulcers suggested an *H. pylori* infection. However, the endoscopic appearance of diffuse erosive gastritis and the negative serum anti‐*H. pylori* antibody result was not typical of peptic ulcer disease. Other causes of gastritis should be considered. Autoimmune gastritis is a well‐known cause of chronic gastritis, but it typically spares the antrum. The moderate erosive gastritis in the antrum was unusual for autoimmune gastritis. Eosinophilic gastritis was unlikely in the absence of eosinophilia or mucosal eosinophil infiltration. Amyloidosis, granulomatous gastritis, and infiltrative tumors were unlikely according to the biopsy results. Additionally, an upper gastrointestinal manifestation of inflammatory bowel disease was unlikely in the absence of lower gastrointestinal symptoms. Collagenous gastritis was possible because of the nodular mucosa, which is an endoscopic feature of collagenous gastritis, but unlikely in the absence of subepithelial collagenous deposition. The working diagnosis was drug‐induced gastritis, particularly olmesartan‐induced gastritis. Because his diabetes mellitus was well controlled and his blood pressure was normal without medication, hypoglycemic and antihypertensive medications were discontinued. His appetite gradually improved during the admission. He was discharged five days after admission. Follow‐up EGD 2 months after discharge revealed that the gastric erosions healed while remaining nodular (Figure 4a,b). His loss of appetite resolved. During the next 10 months, he remained in his usual state. His weight increased to 70 kg 6 months after withdrawing olmesartan. Mosapride and misoprostol were discontinued, and olmesartan was inadvertently readministered for hypertension. Two months after resuming olmesartan treatment, EGD revealed multiple short linear erosions in the antrum (Figure 4c), although the severity was milder than at the initial examination. He had a mild loss of appetite. Therefore, the olmesartan treatment was discontinued. Healing of the gastric erosions and nodular mucosa was confirmed by EGD 1 year later (Figure 4d). He was diagnosed with olmesartan‐induced gastritis and was instructed to avoid all ARBs. Amlodipine was used to treat his hypertension.

## DISCUSSION

This case illustrates a rare adverse reaction of olmesartan on the stomach.

Olmesartan‐induced enteropathy was first reported in 2012.^1^ It is characterized by severe chronic diarrhea and weight loss, occurring months to years after drug initiation. Although the most common cause of ARB‐induced enteropathy is olmesartan, it has been reported for other ARBs, including telmisartan, irbesartan, valsartan, losartan, and eprosartan.^2^ Gastrointestinal toxicity might be a class effect rather than an olmesartan‐specific one. Therefore, we instructed the patient to avoid all ARBs. However, the mechanisms underlying the gastrointestinal toxicity are not well understood. It has been suggested that ARBs inhibit transforming growth factors or lead to an imbalanced activation of angiotensin II type 2 receptors by blocking angiotensin II type 1 receptors, thereby causing immune‐mediated inflammation.^2^, ^3^ ARB‐induced enteropathy can involve the stomach, and gastritis develops 6 months to 12 years after administration.^3^, ^4^, ^5^, ^6^ Gastric histologic lesions were identified in 73% of 11 patients with ARB‐induced enteropathy.^7^ The histological findings were collagenous gastritis, lymphocytic gastritis, eosinophilic gastritis, chronic active gastritis, and metaplastic atrophic gastritis.^1^, ^7^ Another study using pathology archives reported the resolution of gastric inflammation after ARB withdrawal in patients with histologic gastritis.^4^ However, ARB‐induced gastritis with no lower gastrointestinal symptoms is rarely reported.^5^, ^6^, ^8^

The patient exhibited several interesting clinical and endoscopic features. First, esomeprazole failed to prevent the relapse of gastric erosion after the re‐administration of olmesartan. Proton pump inhibitors, including esomeprazole, are the mainstay of treatment for acid‐related diseases and are used to prevent and treat gastroduodenal ulcers induced by nonsteroidal anti‐inflammatory drugs. This raises concerns that olmesartan‐induced gastritis has been overlooked as a proton pump inhibitor‐refractory ulcer of unknown etiology. Second, a clinical response was observed within 1 week after the cessation of olmesartan. Such rapid clinical improvement has been described in previous case reports of olmesartan‐induced gastritis.^5^, ^6^ Epigastric pain began to improve within a week,^6^ and vomiting stopped within 2 weeks after ARB withdrawal.^5^ Similarly, in ARB‐induced enteropathy, clinical improvement typically occurs within a week.^9^ The rapid clinical response, which occurs within 1 or 2 weeks after olmesartan withdrawal, might be a characteristic of olmesartan‐induced gastritis. When EGD showed a mild relapse 2 months after resuming olmesartan treatment, the patient's appetite loss was mild. This suggests that olmesartan‐induced gastritis developed gradually over several months. Finally, endoscopic findings of multiple erosions in the antrum and nodular mucosa of the corpus were characteristic. These changes may provide clues to the correct diagnosis. Current knowledge regarding the endoscopic features of olmesartan‐induced gastritis is limited due to its rarity. Roughened mucosa and erosions in the gastric antrum were described in olmesartan‐induced gastritis.^3^ Multiple linear ulcers were reported in olmesartan‐induced collagenous gastritis.^8^ We performed five biopsies from the stomach and did not find subepithelial collagen deposition; however, the diagnosis of collagenous gastritis cannot be completely ruled out because this case had an endoscopic appearance similar to collagenous gastritis, in which the distribution of subepithelial collagen deposition can be patchy.^10^

In conclusion, we reported a rare case of olmesartan‐induced gastritis without lower gastrointestinal symptoms. The suggested characteristic features were as follows: 1) gastritis developing after several months to years following drug initiation, 2) rapid clinical improvement following olmesartan withdrawal, and 3) endoscopic appearance of antral‐dominant erosive gastritis and nodular mucosa. Endoscopists should be aware of olmesartan as a cause of gastritis.

## CONFLICT OF INTERESTS STATEMENT

None.

## ETHICS STATEMENT

N/A

## Supporting information



Figure S1 The endoscopic appearance of the stomach was observed a year prior to the current presentation. Atrophic gastritis was observed in the esophagogastroduodenoscopy performed at a clinic a year before the current presentation.

Figure S2 Gastric bleeding. Dieulafoy lesion with oozing bleeding in the lesser curvature of the middle corpus, which was not the site of the biopsies.

TABLE S1 Laboratory data.
