# Supplementary figures and images for: Olmesartan‐induced gastritis with no lower gastrointestinal symptoms: A case report
Source: DEN Open. 2025 Apr 29;6(1):e70124. doi: 10.1002/deo2.70124 (PMC12038180; doi:10.1002/deo2.70124)

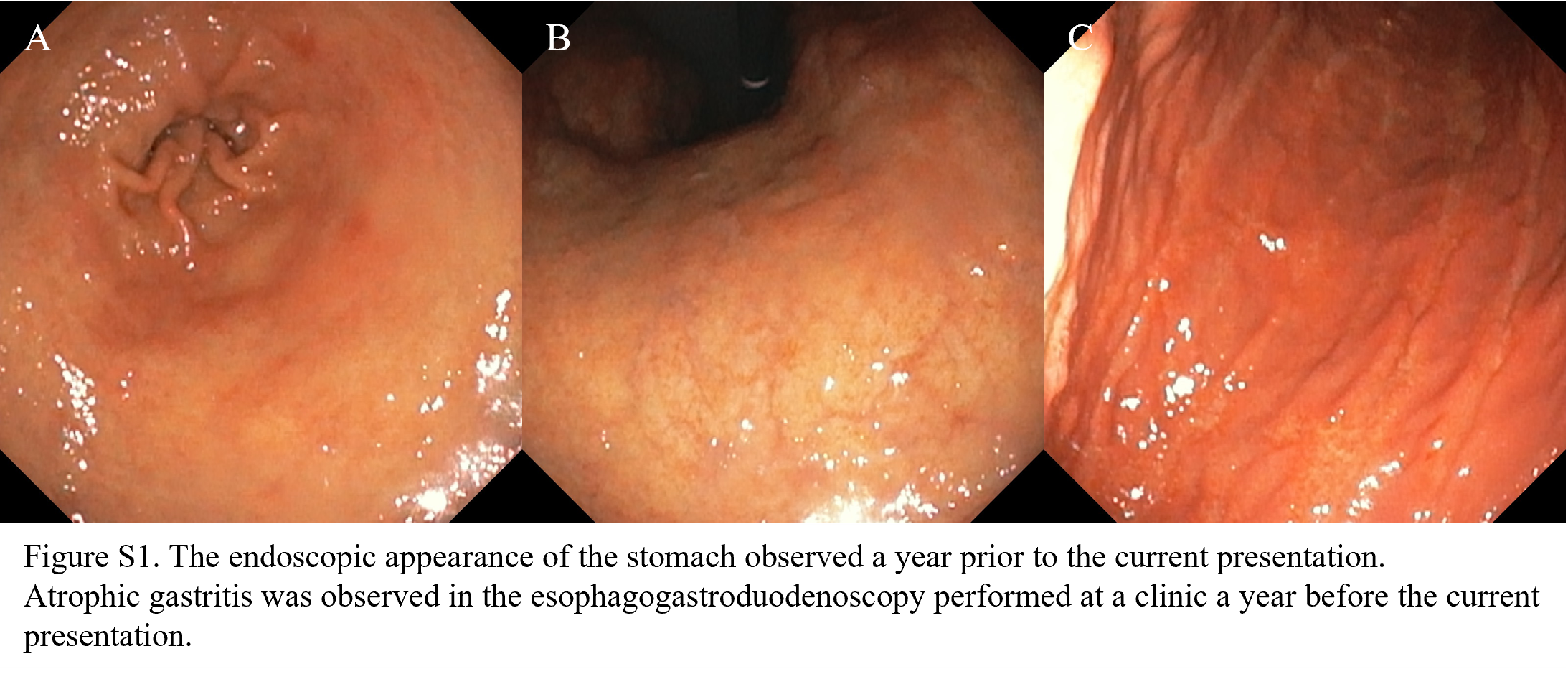

Supplement: Supplementary file 1 — Figure S1 The endoscopic appearance of the stomach was observed a year prior to the current presentation. Atrophic gastritis was observed in the esophagogastroduodenoscopy performed at a clinic a year before the current presentation. [file DEO2-6-e70124-s001.tif]

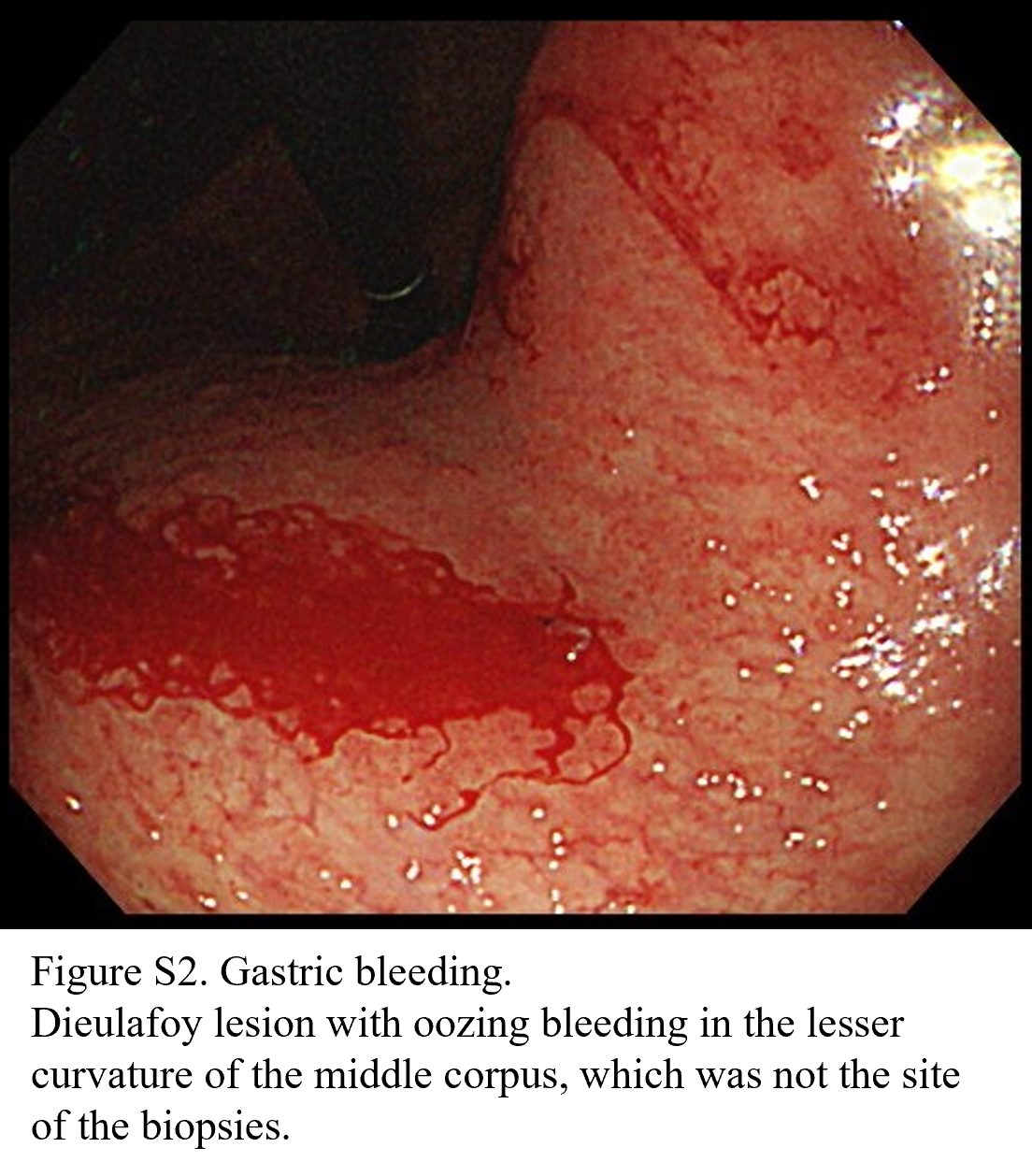

Supplement: Supplementary file 2 — Figure S2 Gastric bleeding. Dieulafoy lesion with oozing bleeding in the lesser curvature of the middle corpus, which was not the site of the biopsies. [file DEO2-6-e70124-s003.tif]
